# Digital Clock and Recall is superior to the Mini-Mental State Examination for the detection of mild cognitive impairment and mild dementia

**DOI:** 10.1186/s13195-023-01367-7

**Published:** 2024-01-02

**Authors:** Ali Jannati, Claudio Toro-Serey, Joyce Gomes-Osman, Russell Banks, Marissa Ciesla, John Showalter, David Bates, Sean Tobyne, Alvaro Pascual-Leone

**Affiliations:** 1 Linus Health, Inc., 280 Summer Street, 10th Floor, Boston, MA 02210 USA; 2 Department of Neurology, Harvard Medical School, Boston, MA USA; 3 Department of Neurology, University of Miami Miller School of Medicine, Miami, FL USA; 4 Department of Communicative Sciences & Disorders, Michigan State University, East Lansing, MI USA; 5 Hinda and Arthur Marcus Institute for Aging Research and Deanna and Sidney Wolk Center for Memory Health, Hebrew SeniorLife, Boston, MA USA

**Keywords:** Alzheimer’s disease, Clock Drawing Test, Cognitive screening, Digital cognitive assessment, Dementia, Mild cognitive impairment, Mild neurocognitive disorder, Mini-Mental State Examination, Neurocognitive disorder, Rey Auditory Verbal Learning Test

## Abstract

**Background:**

Disease-modifying treatments for Alzheimer’s disease highlight the need for early detection of cognitive decline. However, at present, most primary care providers do not perform routine cognitive testing, in part due to a lack of access to practical cognitive assessments, as well as time and resources to administer and interpret the tests. Brief and sensitive digital cognitive assessments, such as the Digital Clock and Recall (DCR™), have the potential to address this need. Here, we examine the advantages of DCR over the Mini-Mental State Examination (MMSE) in detecting mild cognitive impairment (MCI) and mild dementia.

**Methods:**

We studied 706 participants from the multisite Bio-Hermes study (age mean ± SD = 71.5 ± 6.7; 58.9% female; years of education mean ± SD = 15.4 ± 2.7; primary language English), classified as cognitively unimpaired (CU; *n* = 360), mild cognitive impairment (MCI; *n* = 234), or probable mild Alzheimer’s dementia (pAD; *n* = 111) based on a review of medical history with selected cognitive and imaging tests. We evaluated cognitive classifications (MCI and early dementia) based on the DCR and the MMSE against cohorts based on the results of the Rey Auditory Verbal Learning Test (RAVLT), the Trail Making Test-Part B (TMT-B), and the Functional Activities Questionnaire (FAQ). We also compared the influence of demographic variables such as race (White vs. Non-White), ethnicity (Hispanic vs. Non-Hispanic), and level of education (≥ 15 years vs. < 15 years) on the DCR and MMSE scores.

**Results:**

The DCR was superior on average to the MMSE in classifying mild cognitive impairment and early dementia, AUC = 0.70 for the DCR vs. 0.63 for the MMSE. DCR administration was also significantly faster (completed in less than 3 min regardless of cognitive status and age). Among 104 individuals who were labeled as “cognitively unimpaired” by the MMSE (score ≥ 28) but actually had verbal memory impairment as confirmed by the RAVLT, the DCR identified 84 (80.7%) as impaired. Moreover, the DCR score was significantly less biased by ethnicity than the MMSE, with no significant difference in the DCR score between Hispanic and non-Hispanic individuals.

**Conclusions:**

DCR outperforms the MMSE in detecting and classifying cognitive impairment—in a fraction of the time—while being not influenced by a patient’s ethnicity. The results support the utility of DCR as a sensitive and efficient cognitive assessment in primary care settings.

**Trial registration:**

ClinicalTrials.gov identifier NCT04733989.

**Supplementary Information:**

The online version contains supplementary material available at 10.1186/s13195-023-01367-7.

## Introduction

Brain disorders cause greater disability than cardiovascular diseases and cancers combined, and according to the projections by the World Health Organization (WHO), by 2030, brain-related disabilities will contribute to half of the global economic burden caused by disability [1]. With dementia as the leading cause of disability among older adults [2] and Alzheimer’s disease (AD) as the most common cause of dementia, forecasts predict that by 2050, the number of individuals with AD and related dementias (ADRD) will reach 13.8 million in the U.S. [3] and 152 million globally [2].

The recent success of clinical trials for treating AD with anti-amyloid agents (lecanemab, donanemab) [4–6] and the approval of two such agents by the US Food and Drug Administration [7, 8] for early-stage AD—mild cognitive impairment (MCI) and mild dementia—highlight the importance of detecting cognitive impairment at early stages. The donanemab study stratified for disease severity as measured by tau load and found highly different results, with the clear strongest benefit in those with less severe disease [6]. These results confirm that the sooner cognitive impairment (CI) is detected, the larger the benefits of treatment in slowing down the trajectory of the patients’ cognitive decline, preventing loss of functional independence, and minimizing impairment in activities of daily living (ADLs) [9].

This is in sharp contrast with the reality of the current status of cognitive screening. The vast majority of CI cases are detected reactively only after the patients or their family members or care partners report cognitive or memory concerns to healthcare providers. Therefore, when most CI cases are detected, patients are further along the trajectory of cognitive decline and likely outside the optimal window for pharmacological [9] or nonpharmacological (lifestyle and psychosocial) [10, 11] interventions. This underlines the importance of shifting current practices in wide adoption of routine cognitive screening for patients above a certain age (e.g., 55 years old).

The need for routine cognitive screening that can detect CI at early stages in primary care cannot be circumvented by providing broad access to blood-based AD biomarkers alone as some have suggested [12–14]. Biomarker levels lack a strong association with the level of cognitive function and thus cannot reliably predict disability [15], which is frequently the patient’s main concern. The partial dissociation between AD biomarker status and cognitive functioning means that neuropsychological assessment is critical for early detection of cognitive impairment. In fact, up to one-third of individuals with a positive biomarker test do not develop dementia and thus may not be suitable candidates for disease-modifying treatments (DMTs) [15]. On the other hand, approximately 20–25% of individuals aged 65 and above develop mild cognitive impairment (MCI) [16, 17], with 10–15% of individuals with MCI progressing to dementia each year [3].

These findings indicate that both cognitive evaluation and biomarker testing are necessary to provide a complete picture of the patient’s brain function, help define the biology of the disease [18], and critically aid in the identification of suitable candidates for DMTs in a timely manner. However, traditional paper-based neuropsychological tests such as the Mini-Mental State Examination (MMSE) and Montreal Cognitive Assessment (MoCA) may not be suitable for routine cognitive screening in primary care because of their lower sensitivity to early stages of cognitive impairment, long completion times, subjective scoring, need for specialized training to administer and interpret the tests, strong influence of educational and racial/ethnic backgrounds, and limited scalability. Furthermore, they provide only a score, with little to no clinical insight for primary care providers about what to do next. Digital cognitive assessments (DCAs) may address these limitations. To do so, DCAs need to be brief (< 5 min), sensitive for early CI detection, reliable, easily administered by nonphysician staff members, and relatively free from educational, linguistic, or cultural biases. They must also fit seamlessly into the clinical workflow of primary care providers (PCPs), including integration into electronic health records (EHRs) [19].

### Digital Clock and Recall (DCR)

One candidate DCA that provides a solution to these obstacles is the Linus Health Digital Clock and Recall (DCR™). The DCR detects subtle signs of cognitive impairment by analyzing an individual’s performance in a combination of clock drawing and word recall tasks to enable early detection. It incorporates and expands on the DCTclock™ [20, 21] with 3-word immediate and delayed verbal recall tests. The DCR represents a machine learning (ML)-enabled implementation of the *Boston Process Approach (BPA)* [22–25] to provide objective insights into patients’ cognitive functions, including verbal and semantic memory, attention and executive function, visuospatial skills, receptive and expressive language, and simple and complex motor skills. Through analysis of the patient’s process of completing the assessment, and not merely the final product, the DCR offers clinicians valuable insights into subtle cognitive deficits. ML algorithms on the generated metrics can then define sensitive scores and predictors for specific risk (e.g., hippocampal volume loss or amyloid deposition in the brain).

Our principal objective in this study was to evaluate the utility of the DCR compared to the commonly used MMSE for the purpose of cognitive screening for CI, where CI comprises MCI and mild dementia likely due to AD. This is because identifying individuals at this level of impairment is crucial for patients’ eligibility for DMTs and maximizing their benefit from therapeutic interventions.

Specifically, we aimed to:

1. Compare the CI classification by the DCR and the MMSE against cohort classifications based on the Rey Auditory Verbal Learning Test (RAVLT) for assessment of verbal episodic memory, the Trail Making Test-Part B (TMT-B) for assessment of executive function, and the Functional Activities Questionnaire (FAQ) for assessment of functional dependence in daily activities. We hypothesized that due to its higher sensitivity, the DCR would have greater accuracy than the MMSE for detecting CI.
2. Evaluate the ability of the DCR to detect CI among individuals who were labeled as cognitively unimpaired by the MMSE (score ≥ 28) but infact had impairment of verbal episodic memory as confirmed by the RAVLT. We also performed the reverse comparison to evaluate the utility of the MMSE to detect cases of CI missed by the DCR. We hypothesized that due to its higher sensitivity, the DCR would be able to detect memory impairment in a relatively higher number of individuals whose true impairment was missed by the MMSE.
3. Compare the influence of demographic characteristics such as race, ethnicity, and level of education on the DCR and MMSE scores. Due to the digital nature and the process-based (BPA) foundation of the DCR, we hypothesized that the DCR would be less influenced by demographic factors relative to the MMSE.

## Methods

### Sample and assessments

We studied 706 participants from the prospective, multisite, and multivisit Bio-Hermes study (age mean ± SD = 71.5 ± 6.7; 58.9% female; 85.1% White; 11.7% Black or African-American; 2.2% Asian; 9.3% Hispanic or Latino; years of education mean ± SD = 15.4 ± 2.7; primary language English), classified by the study organizers into three cohorts based on clinical diagnosis of MCI or dementia verified through medical records or the results of selected cognitive and functional assessments including the MMSE, RAVLT, and the FAQ (See the Supplementary materials for details of the Bio-Hermes study visit schedule, protocol, cohort classification criteria, and assessments): cognitively unimpaired (CU; *n* = 360), mild cognitive impairment (MCI; *n* = 234), or probable Alzheimer’s dementia (pAD; *n* = 111) [26]. The Bio-Hermes study, organized by the Global Alzheimer’s Platform (GAP), was an effort to collect blood and digital biomarkers from a large, racially and ethnically diverse sample of participants at various levels of cognitive function [27]. Data from the Bio-Hermes study will be publicly available on the Alzheimer's Disease Data Initiative website in the future. Participants in this study performed a battery of neuropsychological tests on the initial visit and questionnaires and the DCR on each of their visits. We included only participants who had a first visit with the DCR, RAVLT, TMT-B (for purposes of cohort definition), and FAQ scores. No follow-up tests were examined, making this a cross-sectional study.

### Digital Clock and Recall (DCR)

The DCR is a self-adminstered, supervised digital cognitive assessment composed of immediate recall, clock drawing (DCTclock), and delayed recall. The immediate and delayed recall components of the DCR consist of 3 words. Patients are verbally presented with 3 words and are asked to immediately repeat them (Immediate Recall). After completing the DCTclock, the patient is then asked to repeat the initial 3 words (Delayed Recall). The Delayed Recall assesses verbal episodic memory, which is the cognitive function particularly impaired at early stages of AD [28–30]. Evaluation of verbal episodic memory is important both for classifying the patient’s current cognitive status and for estimating the likelihood of the patient’s progression to dementia over the subsequent decade [31, 32]. The DCTclock is composed of a Command Clock task followed by a Copy Clock task. In Command Clock, the task is to draw an analog clock from memory with hands set to “10 after 11,” whereas Copy Clock involves copying an already-drawn clock set to the same time. Participants were allowed to take the DCR only once per visit.

A key advantage of the DCTclock is its ability to assess the various cognitive and graphomotor functions that are involved in the process of clock drawing, including drawing efficiency, speed of information processing, simple and complex motor skills, and visuospatial reasoning [20, 33]. ML-enabled scoring provides nuanced measures of motor, cognitive, and time-based performance that are not captured by traditional, visually scored pen-and-paper clock drawing tests (CDTs) or digitized CDTs that rely only on the outcome of the test [20, 33, 34]. These measures enable the detection of subtle preclinical signs of cognitive deficit and the classification of the CI subtype [35].

### Scoring of the immediate and delayed recall

There is no time limit for the Immediate and Delayed Recall tasks. Each word recalled correctly in the Delayed Recall contributes one point (for a maximum of 3 points) toward the total DCR score. The Immediate Recall does not directly contribute to the overall DCR score. However, it is important to review the patient’s Immediate Recall performance to assess potential concerns regarding the patient's hearing, attention, immediate/short-term memory, or executive function. The DCR records the patient’s voice response following the three-word prompt separately for Immediate and Delayed Recall. These recordings are converted to text representations through automated speech recognition and are then compared against the prompted words to calculate accuracy. Internal validation of the automatic speech recognition in the DCR algorithm compared to a human transcriber has shown a 95% recognition accuracy.

### Scoring of the DCTclock

The DCTclock contributes up to 2 points to the overall DCR score. The design and implementation of the DCTclock data analysis engine have been previously reported in detail [20, 33]. Briefly, the measures that are derived from the DCTclock are summarized in a single summary score out of 100 with cutoff scores of < 60, 60–74, and ≥ 75 contributing 0, 1, and 2 points, respectively, to the total DCR score. The DCTclock includes four Command and Copy Clock composite scales, each composed of 22 subscales that evaluate various aspects of the clock drawing process: drawing efficiency, speed of information processing, simple and complex motor skills, and visuospatial reasoning [20, 33, 35]. Out of 1891 DCR tests performed across visits in the original data set, only 4% of the DCTclocks were unanalyzable. More than half of those were not from participants’ first DCR tests, which are the tests evaluated here. No unanalyzable tests were included in this work.

### Scoring of Digital Clock and Recall

**Fig. 1:**
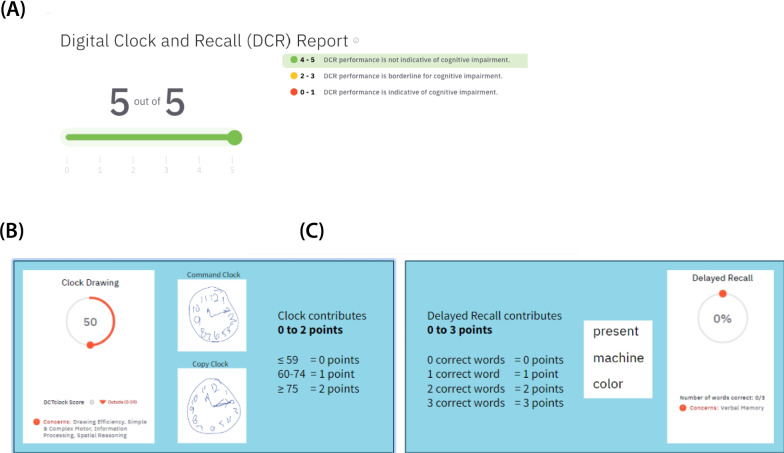
Scoring of the DCR (**A**), DCTclock (**B**), and Delayed Recall (**C**)

The total DCR score is a combination of the DCTclock and the Delayed Recall scores and is presented as 0–5 (Fig. 1A). The DCTclock and the Delayed Recall contribute 0–2 points and 0–3 points to the DCR score, respectively (Fig. 1B, C). The DCR score is represented as Green (DCR score 4–5), Yellow (DCR score 2–3), or Red (DCR score 0–1). A Green DCR score means no indication of CI was detected. Individuals with a the Yellow score are considered borderline for CI. The Yellow DCR score identifies patients who are at the earliest stages of CI and, therefore, may benefit the most from actionable recommendations such as improving their brain health-related lifestyle and psychosocial factors. Patients with a Red DCR score require the most attention because their performance indicates they are likely to have CI and would benefit from referral to specialized services for further evaluation and workup.

The overall scoring method (0–2 points from the clock test and 0–3 points for the delayed recall) is similar between the DCR and the Mini-Cog©. However, the total scores of 0, 1, and 2 on the Mini-Cog indicate a higher likelihood of cognitive impairment (and dementia in many cases), whereas DCR scores 0–3 indicate at least some degree of cognitive impairment (yellow or red) while DCR scores 4–5 (green) are not indicative of cognitive impairment. This is because a loss of 2 out of 5 points in the DCR can occur in three ways: (1) a score of 0 out of 2 in the DCTclock—a likely indication of impairment in at least some of the various cognitive domains assessed by the DCTclock including executive function and visuospatial skills; (2) failure to recall 2 of the 3 words—a likely indication of verbal episodic memory; or (3) loss of 1 point from the DCTclock and 1 point from the delayed recall—a likely indication of subtle or mild impairment in a mixture of some of the domains assessed by the DCTclock and verbal episodic memory. Scores 0, 1, or 2 on the DCR indicate an even greater likelihood of impairment in these cognitive domains.

### Cohort classification

The Bio-Hermes study protocol includes cohort classification based on a mix of expert evaluation and neuropsychological assessment results that included the MMSE. To avoid circularity in our comparison of the DCR and the MMSE, we devised an objective, rules-based cohort classification scheme based on memory, executive, and/or functional impairment as assessed by the RAVLT, TMT-B, and FAQ, respectively. Verbal episodic memory impairment on the RAVLT was defined as a long delay score ≥ 1 standard deviation (SD) below age-adjusted means [36]. Executive dysfunction on the TMT-B was defined as completion time ≥ 1 SD above the mean of age-adjusted population means [37]. Functional impairment on the informant-reported FAQ was defined as an FAQ score ≥ 6 [38, 39]. As a final measure, given our interest in the early detection of cognitive decline, we excluded participants with an FAQ > 9 (as these could be considered to be further into the disease progression, including moderate-to-severe dementia).

**Fig. 2:**
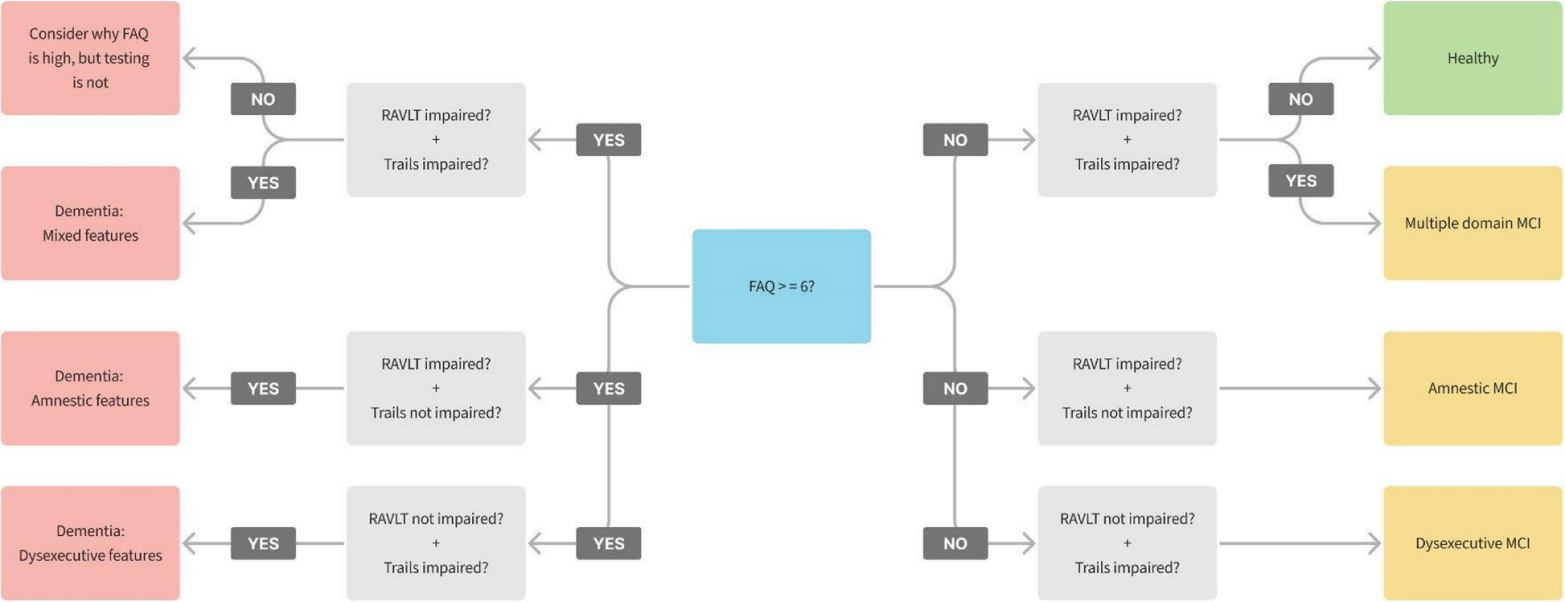
Cohort classification scheme based on the FAQ, RAVLT, and TMT-B scores. Starting with evaluating functional impairment (i.e., FAQ score ≥ 6), the decision tree considered verbal-memory (RAVLT) and executive (TMT) impairment. Impairment was defined as at least 1 standard deviation away from the age-adjusted mean in the direction of worse performance (e.g., slower TMT)

The rules-based classification scheme produced the following cohorts (Fig. 2): Cohort 1 (healthy), Cohort 2 (single-domain amnestic MCI; aMCI), Cohort 3 (multiple-domain amnestic MCI; mdMCI), Cohort 4 (dysexecutive or non-amnestic MCI; naMCI), and Cohort 5 (probable mild ADRD). Cohorts 2 through 5 were then combined together in order to generate the final cognitively impaired (CI) cohort that was the target of our classification analyses.

### Analyses

The analyses addressed three goals:

1. Comparing the overall CI detection (rules-based Cohort 1 [healthy] vs. the other four cohorts) based on the DCR and the MMSE;
2. Comparing the classification accuracy and sensitivity of the DCR vs. the MMSE for detecting memory impairment as confirmed by impairment on the RAVLT delayed recall (long delay score ≥ 1 standard deviation (SD) below age-adjusted means [36]);
3. Comparing the influence of Race (White vs. Non-White), Ethnicity (Hispanic vs. Non-Hispanic), and Educational level (high [≥ 15 yrs] vs. low [< 15 yrs]) on DCR and MMSE scores.

To address the first goal, we used a machine-learning (ML) classification approach. We first split the data into training (70%) and testing (30%) sets to avoid overfitting. We ensured that training and test sets were balanced for the distributions of the respective target cohorts for each goal. The rules-based classification scheme, when applied to this dataset, produced an imbalance in the distribution of the resulting categories (e.g., more MCI participants than healthy controls). When the class imbalance is severe, improperly trained ML models learn to predict the majority class only because this optimizes their accuracy. To mitigate this issue, we used standard upsampling procedures on the training set, where participants were randomly sampled with replacement until all classes matched the frequency of the majority class. This procedure was not performed on the test set, which retained the distribution of the original categories that was representative of the sample. We then trained random forest (RF) models to classify training set cohorts and evaluated model performance using the test set. All RF models were tuned to use the least number of features per iteration, for as long as the out-of-bag error estimates were over 0.001 and the number of trees was within 500.

To examine the potential variability of performance across train and test splits, we repeated the modeling procedure 200 times, each time using a different, random train-test partition of the data. Each of these splits followed the rules described above. We then computed the AUC for each iteration for the DCR and MMSE models. We report the distribution and central tendencies of the AUC for each model. Unlike *k*-fold cross-validation, this procedure allowed splits from different iterations to be similar to one another, providing a more nuanced sense of the influence of variability in our data [40]. We report AUCs and accuracy metrics (sensitivity, specificity, balanced accuracy) based on optimized decision thresholds given by the Youden statistic (i.e., the best balance between sensitivity and specificity). We report summary statistics for the AUCs across these iterations.

For Goal 1, we compared models that included either DCR features only, MMSE total score only, demographics only (age, sex, and years of education), and a model that used all these sources of information. This final model was implemented to act as an upper bound of classification performance for this dataset to contextualize the performance of the other models. DCR predictor features consisted of all age-scaled subscores that compose the DCTclock (no composite scores; see Scoring the DCTclock for details) and the delayed recall score.

For Goal 1, we thresholded the MMSE total score at 28, reflecting the way this cutoff is often used in clinical settings to rule out cognitive impairment [41–43]. For Goal 2, we calculated the proportion of individuals with a DCR score ≤ 3 among the subset of individuals who had an MMSE score ≥ 28 but in fact had memory impairment as confirmed by delayed recall performance on the RAVLT. Conversely, we calculated the proportion of individuals with an MMSE score < 28 among the subset of individuals who had a DCR score ≥ 4 but had memory impairment as confirmed by the RAVLT.

We performed three analyses to address the third goal. First, we compared the DCR and MMSE scores in White vs. Non-White, Hispanic vs. Non-Hispanic, and High vs. Low education groups using two-sample Wilcoxon rank sum tests. Second, we performed two Poisson regressions, each predicting either DCR or MMSE scores from a set of features that included race, ethnicity, education, sex, age, and cohort status (cognitively unimpaired or impaired). Third, we compared the degree of demographic bias using a bootstrapped procedure. For each demographic characteristic separately, we sampled 100 individuals from each group (e.g., Hispanic and Non-Hispanic) with replacement 5000 times. On each of the 5000 iterations, we performed a linear model for DCR and MMSE separately, where the model predicted the previously computed scaled score of the test using predictors for race, ethnicity, years of education, sex, age, and cohort status. The coefficients for the respective demographic factor of interest were used to calculate the mean difference between demographic groups (i.e., the bias of the test while accounting for other demographics). We then calculated the difference between the biases in the two tests (DCR* minus* MMSE). Based on the resulting 5000 differences of bias, we calculated the 95% confidence intervals to establish whether either test had an overall smaller difference between demographic groups (i.e., distribution of differences significantly above or below zero). This procedure allowed us to evaluate if either test had a significantly smaller bias relative to the other test.

### Tools used

Analyses were performed with the R statistical programming language [44] (v4.1.3). The packages used in this work included Tidyverse [45] (v2.0.0), corrplot [46] (v0.92), lme4 [47] (v1.1-33), gt [48] (v0.9.0), randomForest (v4.7-1.1) [49], pROC (v1.18.0) [50], and caret (v6.0-94) [51].

## Results

### Cohort counts

The rules-based cohort classification among a total of 706 individuals with available and eligible data resulted in a total of 331 (46.8%) participants in Cohort 1 (Cognitively Unimpaired), 176 (24.9%) in Cohort 2 (single-domain amnestic MCI; aMCI), 61 (8.6%) in Cohort 3 (multiple-domain MCI; mdMCI), 71 (8.6%) in Cohort 4 (non-amnestic MCI; naMCI), and 59 (8.3%) in Cohort 5 (probable probable mild ADRD). Cognitively impaired and unimpaired cohorts for the first set of analyses were created by grouping Cohorts 2 through 5 into a single cognitively impaired group. The rules-based cohort approach resulted in 331 (46.6%) impaired and 374 (52.9%) unimpaired individuals. One participant did not have a valid reported score. Demographics for each cohort are provided in Table 1.

**Table 1:** Demographic information for each of the resulting cohorts. *χ*^2^ refers to a chi-squared test for equality of proportions. The *T*-values provided are for independent-samples *T*-tests. *W*-values refer to the statistics for Wilcoxon rank sum tests

	Unimpaired % or Median (SD)	Impaired (CI) % or Median (SD)	Comparison test
Total *N*	331	374	
Sex (Females)	65%	52%	*χ*^2^ = 11.58, *p* < 0.001
Ethnicity (Hispanic)	6%	11%	*χ*^2^ = 4.37, *p* < 0.05
Race (White)	91%	79%	*χ*^2^ = 21.34, *p* < 0.0001
Age in years	71.9 (6.8)	71.2 (6.6)	*T* = 1.23, *p* = 0.21
Years of education	16 (2.5)	16 (2.7)	*W* = 69.94, *p* < 0.01
MMSE	29 (1.7)	27 (2.7)	*W* = 38.22, *p* < 0.00001
RAVLT Total Score	8 (2.3)	4 (3.0)	*W* = 106.32, *p* < 0.0001
Trails B Duration	105 (42.3)	151 (36.7)	*T* = − 10.37, *p* < 0.00001
PET Aβ Positivity	23%	36%	*χ*^2^ = 12.91, *p* < 0.001

### Cognitive impairment classification by the DCR vs. MMSE

**Fig. 3:**
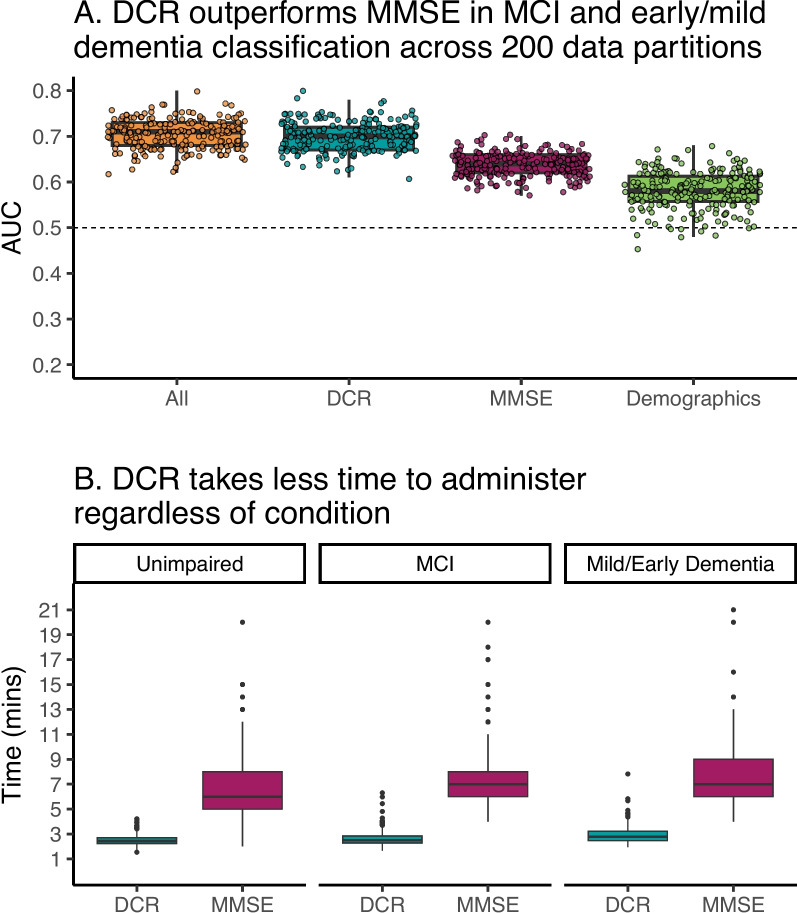
**A** AUCs for 200 iterations of the binary mild cognitive impairment (MCI) and mild/early dementia classification models. On each iteration, we randomly split the data into train/test sets using the same distribution matching and upsampling procedure. For each split, we then fitted the DCR and MMSE models as before and stored the resulting AUC. On average, the AUC for the DCR-based model (median = 0.70, SD = 0.03) was significantly greater than that for the MMSE (median = 0.63, SD = 0.02; paired permutation *p* < 0.0001) and was as good as the model inclusing all the sources of information (median = 0.7, SD = 0.032) including the DCR, the MMSE, and demographics. The dashed line represents 50% (chance level) classification accuracy. **B** The DCR took significantly less time to administer regardless of cohort (log-normal regression, *p* < 0.0001) and was less variable (DCR SD = 0.53; MMSE SD = 2.43)

For our first goal, we evaluated which test would perform better at classifying general cognitive impairment, defined as all impaired cohorts from the rules-based classification. We examined performance as a function of random train and test splits by repeating the model-fitting procedure from Goal 1 using 200 different random train-test splits (see “Methods” for details). In this way, it is possible to estimate the extent to which the selection of a single random train-test split influences the model and would result in a poorly performing model when faced with real-world data. Both median sensitivity and negative predictive value (NPV) were higher for the DCR (sensitivity = 0.67, SD = 0.04; NPV = 0.62, SD = 0.03) than for the MMSE (sensitivity = 0.57, SD = 0.03; NPV = 0.59, SD = 0.02) and demographics (sensitivity = 0.51, SD = 0.06; NPV = 0.52, SD = 0.03). In contrast, median specificity and positive predictive value (PPV) were higher for the MMSE (specificity = 0.69, SD = 0.03; PPV = 0.68, SD = 0.03) than for the DCR (specificity = 0.61, SD = 0.04; PPV = 0.66, SD = 0.02) and demographics (specificity = 0.56, SD = 0.07; PPV = 0.59, SD = 0.03). However, as seen in Fig. 3A, the AUC for the DCR (median = 0.70, SD = 0.030) was significantly higher than the AUC for the MMSE (median = 0.63, SD = 0.03) across partitions (paired permutation on median AUC differences, *p* < 0.0001; 5000 iterations), whereas the demographics-only model performed the worst (median = 0.48, SD = 0.03). The model with all features also had a median of 0.7 (SD = 0.032), indicating that the DCR alone was as good as all sources of information combined. These results add confidence to the observed better performance of the DCR in detecting cognitive impairment via a performance variability estimation that is rarely reported in this kind of study. Given the probability of low scores in older cognitively unimpaired individuals [52], we replicated this analysis using a more stringent - 1.5 SD threshold on the RAVLT during cohort definition. The results are shown in Supplementary Figure S1 and Supplementary Table S1. The relative differences in AUC among tests were similar to those obtained with a - 1 SD threshold.

The higher performance of DCR is accompanied by a consistently lower and less variable adminitration time, as shown in Fig. 3B (DCR: median = 2.5 min, SD = 0.53; MMSE: median = 6 min, SD = 2.43). A log-linear model estimating time to complete based on the interaction between test (DCR or MMSE) and cohort (unimpaired, MCI, mild/early dementia) showed that (1) the MMSE administration took significantly longer (*t* = 51.09, *p* < 0.0001) and (2) both impaired cohorts tended to be slower overall in completing these tests (MCI: *t* = 2.04, *p* < 0.05; dementia: *t* = 6.84, *p* < 0.0001). The difference between test administration times did not significantly vary as a function of cohort status.

### Sensitivity for detecting memory impairment by the DCR vs. the MMSE

**Fig. 4:**
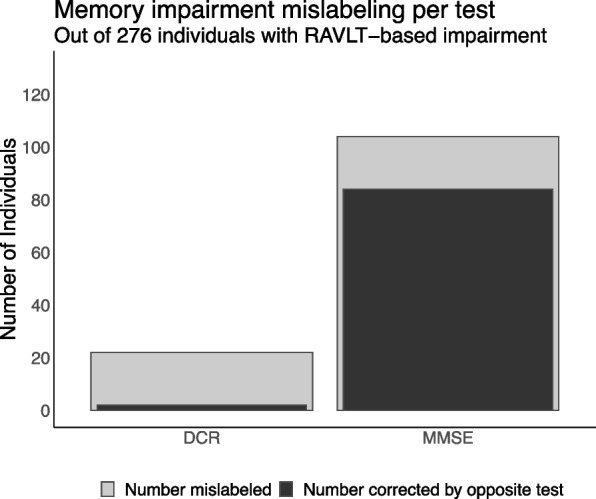
Threshold-based classification of RAVLT-confirmed verbal memory impairment shows that the DCR commits substantially fewer misclassifications than the MMSE (light gray) and *rescues* more of the misclassifications done by the MMSE than vice versa (dark gray). Memory impairment was defined as delayed recall performance on RAVLT at or more than 1 SD below the age-normed mean. Impairment on the DCR was defined as a score of 3 or below, whereas impairment on the MMSE was defined as a score below 28

For the second goal, we evaluated the degree to which each cognitive assessment missed identifying participants with memory impairment (a total of 276 individuals) using a simple score threshold and to what degree the tests disagreed with each other (Fig. 4). A total of 104 individuals were labeled as cognitively unimpaired by the MMSE (score ≥ 28) but were impaired in their RAVLT delayed recall performance (i.e., 37.6% misclassified). However, DCR scores ≤ 3 identified 84 (80.7%) of those missed individuals (i.e., corrected or *rescued*). In contrast, only 22 individuals were labeled as cognitively unimpaired by the DCR (score ≥ 4) but were found to be impaired in their RAVLT delayed recall performance (i.e., 10.6% misclassified), among whom an MMSE score < 28 identified only 2 individuals (10%). In short, compared with the MMSE, the DCR missed far fewer individuals confirmed by the RAVLT to be memory-impaired, and it also recovered a much higher proportion of the cases missed by the MMSE than vice versa.

### Influence of diversity and education level on the DCR and MMSE

**Fig. 5:**
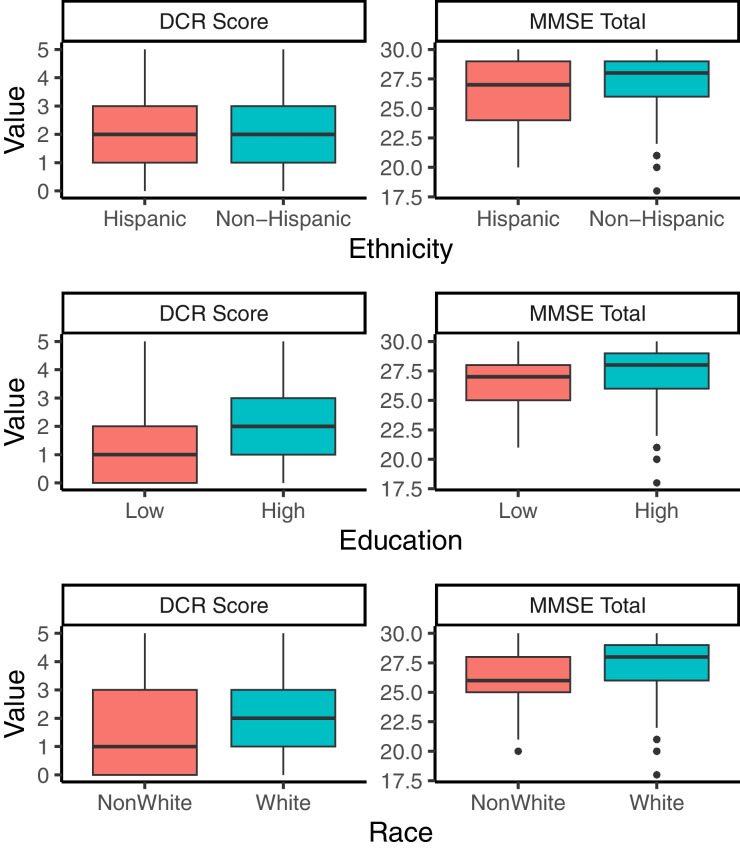
Influence of ethnicity, education, and race on DCR and MMSE scores. DCR scores were not significantly different between Hispanics and Non-Hispanics. A bootstrapping procedure showed that the bias (i.e., differences between groups per demographic) was always lower for the DCR

After evaluating the classification performance of these tests, we wanted to examine their bias due to different demographic factors. Figure 5 displays the results as boxplots for each demographic group and test. The MMSE (but not the DCR) performance was significantly different between Hispanic and non-Hispanic individuals (*V* = 16418, *p* < 0.01). As expected, individuals with higher education outperformed those with lower education in both tests (all *p*’s < 0.0001). A Poisson regression with predictors for sex, age, education, race, ethnicity, and cohort status showed that neither DCR nor MMSE score was significantly different between Hispanic and Non-Hispanic individuals (both *p*’s > 0.05), but both tests showed a significant influence of education (*p*’s < 0.05). In terms of race subgroups, the DCR scores were significantly different (*p* < 0.05) while the difference in MMSE scores did not reach significance (*p* = 0.09) when accounting for cohort status. However, differences in the existence of statistical significance do not by themselves show which test is less biased compared to the other test. To address that question directly, we conducted a comparative bootstrapping procedure (see “Methods” for details) that showed the bias (i.e., the overall difference between demographic subgroups based on the coefficients of a linear model) due to ethnicity was significantly larger for the MMSE than for the DCR (median scaled bias difference = 0.44 larger for the MMSE, two-sided 95% CI = 0.12–0.75). In contrast, the bias due to either race (median scaled bias difference = 0.06 larger for the MMSE, two-sided 95% CI = - 0.25–0.35) or education (median scaled bias difference = 0, 95% CI = - 0.04–0.02) was not significantly different between the two tests.

## Discussion

The present results indicate that the DCR has greater sensitivity and overall accuracy than the MMSE for detecting and classifying cognitive impairment (CI). In addition, we found that the DCR has greater sensitivity than the MMSE for detecting verbal episodic memory impairment as confirmed by the RAVLT, which is a hallmark of early stages of AD. Thus, the DCR has more sensitivity for early detection of AD than the MMSE. Moreover, the DCR has a substantially shorter administration time. Finally, the DCR score is significantly less biased by an individual’s ethnicity and demographic factors overall than the MMSE.

Classification models trained on features from the DCR substantially enhance the capabilities of this brief test relative to traditional tests. DCTclock, as a next-generation assessment analyzing the *process* of task completion while performing a neuropsychological assessment (the *Boston Process Approach*), compared to focusing on the outcome only, has previously demonstrated high sensitivity and classification accuracy for detecting mild cognitive impairment [20, 33–35]. The present results build upon those DCTclock findings by demonstrating the superiority of the DCR, which combines the DCTclock with immediate and delayed verbal recall, for detecting and classifying CI compared to the MMSE. Our findings also show that relying on the MMSE for cognitive screening can result in missing nearly one-third (32%) of patients at earlier stages of amnestic cognitive impairment, for whom initiating treatment and behavioral interventions hold the most promise. In contrast, the DCR was able to identify cognitive impairment in more than 80% of those misclassified by the MMSE, enabling early interventions that can substantially change the trajectory of those patients’ brain health [6, 9, 11].

We purposefully chose a higher score cutoff for the MMSE (≥ 28) than some of the lower cutoffs used in other studies (e.g., ≥ 27 or ≥ 26) to be the most conservative, i.e., to allow the MMSE to have the best chance at outperforming the DCR by flagging a larger number of scores (< 28 rather than, e.g., < 27 or < 26) as cognitively impaired. The finding that, despite such a conservatively chosen score cutoff for the MMSE, the DCR had higher sensitivity and NPV than the MMSE for identifying cognitive impairment indicates that the DCR is a superior test for cognitive screening and is better at detecting individuals at earlier stages of cognitive impairment, many of whom may be missed by the MMSE. Higher specificity and PPV of the MMSE likely reflect the fact that by the time an individual receives a relatively low score on the MMSE, s/he is more likely to be further along the trajectory of cognitive decline and therefore more likely to be actually impaired.

Previously published results using the MMSE to classify MCI, AD, and vascular cognitive impairment reported higher performance values [43, 53–55]. Those previous studies may have overestimated the predictive power of the MMSE. This is potentially due to common issues in machine learning that inflate accuracy estimates, including limited sample sizes, severe class imbalance, or advantageous class comparisons that are not representative of the base rates (e.g., comparing a large cognitively unimpaired group to a small group with dementia instead of the more difficult comparison to MCI). The results detailed herein do not suffer from these limitations.

Several studies have documented significant biases in the MMSE due to race, ethnicity, education level, and socioeconomic status, which often necessitate different scoring criteria for certain demographic groups [56–62]. The present results showed that the DCR is significantly less influenced than the MMSE by ethnicity, indicating the greater utility of the DCR compared to the MMSE for cognitive screening in diverse populations. Given the disproportionate prevalence of ADRD among ethnic minorities, which can amplify existing socioeconomic disparities and lead to worse health outcomes in these populations [62], the deployment of DCAs that are less biased by demographic factors and can maintain their utility across ethnic groups gains additional importance. Such accessibility can also enable the development of services and resources for care partners that are more consistent with the needs and circumstances of the local population [63].

### Additional considerations and future directions

Based on the National Institute on Aging—Alzheimer’s Association (NIA-AA) Research Framework for the biological definition of AD [18], incorporating biomarker data into cognitive classifications in future studies will allow for establishing the utility of the DCR for identifying CU, MCI, and dementia groups who are amyloid- and/or tau-positive or negative. Such an approach will enable a more precise approach to patient triage and investigating pharmacological treatments that target specific pathways in the pathophysiological process of AD and in the appropriate individuals.

Although the sample size used in the present study was relatively large and diverse, there remains the possibility that the composition and characteristics of participants in the Bio-Hermes study are not adequately representative of the wider population of individuals at risk, which constitutes the growing aging population across linguistic, cultural, and socioeconomic groups. Replications of the present findings among non-English speakers and individuals hailing from diverse geographic regions and cultures are crucial for the successful adoption of DCAs such as the DCR among PCPs and patients and for confirming the external validity of these results in clinical settings.

While the use of comprehensive neuropsychological evaluation as a reference was beyond the scope of the present study, a more detailed characterization of patients’ cognitive phenotypes may provide a more nuanced picture of their cognitive deficits, against which the utility of the DCR and its subscores can be evaluated.

## Conclusions

Our results indicate that the DCR is a superior cognitive screening test to the MMSE in primary care settings where early detection of MCI is critical. Compared with the MMSE, we found that the DCR has greater sensitivity and higher overall accuracy for detecting and classifying cognitive impairment and less demographic bias in a substantially shorter administration time (~ 3 min for the DCR compared to ~ 7–12 min for the MMSE). In addition, the DCR is easier to administer by nonphysician staff members and offers objective and automatic scoring. Thus, the DCR can more readily fit into the PCPs’ routine clinical workflow, be completed along with other vital signs while the patient is waiting for their PCP, and alleviate the time pressure constraints experienced by the PCPs due to busy schedules and brief clinical visits. Moreover, the digital platform on which the DCR is administered allows for easy integration of the results into the patient’s EHR as structured data that can be tracked longitudinally.

The DCR can increase the accuracy and efficiency of clinical decision-making, patient triage, and treatment planning for patients at earlier stages of cognitive impairment, thereby providing a larger window of opportunity for their benefitting from approved treatments and for researchers’ investigation of novel therapeutics.

### Supplementary Information

**Additional file 1: Supplementary Figure S1.** Distribution of Area Under the receiver operating characteristic Curve (AUC) for each model based on a 200-iteration bootstrapped procedure. This version uses a more stringent RAVLT threshold of -1.5 SD in order to determine amnestic components of MCI. Relative differences are the same as in the results using the original -1 SD RAVLT threshold. **Supplementary Table S1.** Classification performance per test using cohorts with a RAVLT threshold of -1 SD. PPV = positive predictive value; NPV = negative predictive value; AUC = area under the receiver operating characteristic curve.

## Data Availability

The data that support the findings of this study were collected as part of the Bio-Hermes study (ClinicalTrials.gov Identifier: NCT04733989) and are governed by the Global Alzheimer’s Platform (GAP) consortium agreement. Data are made available via the Alzheimer’s Disease Data Initiative (ADDI) Workbench. All requests for data access should be made directly to GAP. The code used to calculate the reported results is available from Linus Health, Inc. upon reasonable request and with the permission of Linus Health, Inc. Usage restrictions apply to the availability of this code, which is not immediately publicly available.
