# Supplementary material for: Digital Clock and Recall is superior to the Mini-Mental State Examination for the detection of mild cognitive impairment and mild dementia
Source: Alzheimers Res Ther. 2024 Jan 2;16:2. doi: 10.1186/s13195-023-01367-7 (PMC10759368; doi:10.1186/s13195-023-01367-7)
Supplement: Supplementary file 1 — Additional file 1: Supplementary Figure S1. Distribution of Area Under the receiver operating characteristic Curve (AUC) for each model based on a 200-iteration bootstrapped procedure. This version uses a more stringent RAVLT threshold of -1.5 SD in order to determine amnestic components of MCI. Relative differences are the same as in the results using the original -1 SD RAVLT threshold. Supplementary Table S1. Classification performance per test using cohorts with a RAVLT threshold of -1 SD. PPV = positive predictive value; NPV = negative predictive value; AUC = area under the receiver operating characteristic curve. [file 13195_2023_1367_MOESM1_ESM.docx]

**Supplementary Material for**

**Digital Clock and Recall is Superior to the Mini-Mental State Examination for the Detection of Mild Cognitive Impairment and Mild Dementia**

Ali Jannati^1,2*^, Claudio Toro-Serey^1^, Joyce Gomes-Osman^1,3^, Russell Banks ^1,4^, Marissa Ciesla^1^, John Showalter^1^, David Bates^1^, Sean Tobyne^1^, Alvaro Pascual-Leone^1,2,5*^

^1^ Linus Health, Inc., Boston, MA, USA

^2^ Department of Neurology, Harvard Medical School, Boston, MA, USA

^3^ Department of Neurology, University of Miami Miller School of Medicine, Miami, FL, USA

^4^ Department of Communicative Sciences & Disorders, Michigan State University, East Lansing, MI, USA

^5^ Hinda and Arthur Marcus Institute for Aging Research and Deanna and Sidney Wolk Center for Memory Health, Hebrew SeniorLife, Boston, MA, USA

* Corresponding Authors: ajannati@linus.health or apleone@linus.health; Linus Health, Inc., 280 Summer 280 Summer Street, 10th Floor, Boston, MA 02210

##

##

##

##

##

##

##

##

##

##

##

##

##

##

##

##

##

##

##

##

**Table of Contents**

[Eligibility Criteria 3](#_heading=h.2xcytpi)

[Bio-Hermes Study Visit Schedule and Protocol 5](#_heading=h.1ci93xb)

[Visit 1 (Screening) 5](#_heading=h.3whwml4)

[Visit 2 (Imaging) 6](#_heading=h.2bn6wsx)

[Visit 3 (Follow-Up) 6](#_heading=h.qsh70q)

[Schedule of Events 6](#_heading=h.3as4poj)

[Cognitive Cohort Classification 7](#_heading=h.1pxezwc)

[Cognitive Assessments 9](#_heading=h.2p2csry)

[Supplementary Results 10](#_heading=h.147n2zr)

##

##

## Eligibility Criteria

| **Eligibility Criteria:** | In general, participants will be age 60 to 85 years (inclusive) with cognitive levels ranging from normal cognition to mild Alzheimer’s disease.  **Inclusion Criteria:**  Participants must meet all the following criteria for entry into the study:   1. Participants must provide written consent in the IRB approved informed consent form or have a Legal Authorized Representative (LAR) provide written consent in the IRB approved consent form on the participant’s behalf; 2. Male or female 60 to 85 years of age (inclusive) at the time of consent; 3. Participants must be willing to undergo an amyloid PET scan within 60 days of signing informed consent; 4. Participants must have a study partner who, in the investigator’s judgement, has sufficient and frequent contact with the participant and is able to provide accurate information regarding the participant’s cognitive and functional abilities; 5. Participants must be willing to comply with all study procedures as outlined in the informed consent, including blood sampling; 6. Fluency in the language of the tests used at the study site; 7. Participants must be willing to be contacted for possible participation in clinical research trials once their participation in this study ends; and 8. Participants must have a Mini-Mental Status Exam (MMSE) score of 20 to 30 inclusive at Screening.   **Exclusion Criteria:**  Participants who meet any of the following criteria will not be eligible for entry into the study:   1. Participants who, in the opinion of the Site Principal Investigator, have serious or unstable medical conditions that would prohibit their completion of all study procedures and data collection; 2. Participants who have serious or unstable medical conditions that would likely preclude their participation in an interventional research trial; 3. Participants who are unable to undergo amyloid PET due to self-reported pregnancy, sensitivity of ligands being used, poor venous access, contraindication to PET, or present or planned exposure ionizing radiation; 4. Participants who have reported or have a known negative amyloid PET scan in past 12 months; 5. Participants with self-reported, untreated conditions such as vitamin B12 or folate deficiency or bladder infections that in the opinion of the Site Principal Investigator could contribute to cognitive impairment; 6. Participants with history of stroke or seizures within 1 year of screening visit; 7. Participants with history of cancer within the past 5 years with the exception of non-melanoma skin cancer or prostate cancer in situ; 8. Participants with known or suspected alcohol or drug abuse or dependence within 1 year of screening visit; 9. Participants who report any current unstable psychiatric symptoms that could interfere with study procedures or impact study data (e.g., uncontrolled depression); 10. Participants who have participated in a clinical trial of any potential disease modifying AD treatment and received active drug within 6 months prior to Visit 1; 11. Participants who have completed clinical or observational study procedures (e.g., imaging, cognitive testing) within 3 months of screening visit; 12. Participants who have any neurological disorder affecting the Central Nervous System (CNS), other than AD, that may be contributing to cognitive impairment (e.g., Parkinson’s disease, other dementias, multiple concussions or seizures) as deemed significant by the Site Principal Investigator; 13. Participants with a Geriatric Depression Scale (GDS) score greater than or equal to 8 at screening visits; 14. Participants with a Rey Auditory Verbal Learning Test- Delayed Recall Score of 1.5 standard deviation above the age-adjusted mean; 15. Participants with known history or self-report to be Human Immunodeficiency Virus (HIV) Positive; 16. Participants weighing less than 110 pounds; 17. Participants that have previously been consented to this study; 18. Participants who are direct employees or family members of direct employees of the participating investigators’ sites; 19. Participants who are direct employees of the Sponsor; 20. Participants who, in the opinion of the investigator, are unable to complete cognitive testing due to inadequate visual or auditory acuity; |
| --- | --- |
| **Study Duration:** | All participants will have at least 1 visit to collect demographic, clinical, and cognitive information. Participants meeting all eligibility criteria will proceed to an imaging visit to complete the amyloid PET brain scan. There will be a follow-up visit to discuss amyloid PET brain scan result. Sites may request to conduct study visits and procedures remotely with written approval from the Sponsor. Remote procedures include but are not limited to use of telehealth. |

## Bio-Hermes Study Visit Schedule and Protocol

All participants completed one screening visit (**Visit 1**) to collect demographic, clinical, and cognitive information; one imaging visit (**Visit 2**) to complete amyloid PET scan; and one follow-up visit (**Visit 3**) to receive results of amyloid PET scan.

All screening and follow-up visits and procedures were conducted at the clinic whenever possible except some that were completed remotely, when necessary, with written Sponsor approval.

### Visit 1 (Screening)

The following procedures were completed during Visit 1:

1. Obtain informed consent

2. Collection of contact and demographic information including, but not limited to, gender, date of birth, race, and ethnicity

3. Collection of recruitment source

4. Collection of vital signs (includes blood pressure, heart rate, respiration rate)

5. Collection of height and weight

6. Review of Inclusion and Exclusion Criteria

7. Medical History including known family history

8. Concomitant Medications

9. Psychometric Assessment (MMSE, RAVLT, FAQ, GDS)

10. Cognivue® Clarity for Cognitive Assessment

11. Linus Platform Tests

12. Biospecimen Collection

### Visit 2 (Imaging)

At Visit 2, participants underwent an amyloid PET brain scan at a designated imaging center. The results of this scan were reviewed at Visit 3 (Follow-up).

### Visit 3 (Follow-Up)

Participants completed a follow-up visit at the site to allow the Site Principal Investigator or other appropriate study staff to disclose and review the results of PET imaging. PET imaging results were disclosed to all study participants unless the participant declined this disclosure after a meaningful discussion with the Site Principal Investigator or designee. The following procedures were completed during this visit:

1. Review of Medical History or other relevant Health Information

2. Review of Concomitant Medications

3. Speech Vitals

4. Biospecimen Collection

5. Disclosure of amyloid PET brain scan results

6. Adverse Event Collection

### Schedule of Events

| **Activity** | **Visit 1** | **Visit 2** | **Visit 3** |
| --- | --- | --- | --- |
| **Visit Window** | **Day 1** | **Day 30**  **(+ 30 days)** | **Day 60**  **(+ 30 days)** |
| Informed Consent | X |  |  |
| Contact and Demographic Information | X |  |  |
| Collection of Recruitment Source | X |  |  |
| Vital Signs | X |  |  |
| Height and Weight | X |  |  |
| Review of Inclusion and Exclusion Criteria | X |  |  |
| Medical History | X |  | X |
| Concomitant Medications | X |  | X |
| MMSE | X |  |  |
| RAVLT | X |  |  |
| FAQ | X |  |  |
| Geriatric Depression Scale (GDS) | X |  |  |
| Cognivue® Clarity for Cognitive Assessment | X |  |  |
| Linus Health Digital Clock and Recall (DCR™) | X |  | X |
| Biospecimen Collection | X |  | X |
| Amyloid PET Scan |  | X |  |
| Amyloid PET Imaging Results Review* |  |  | X |
| Adverse Event Collection |  |  | X |

*Participants with PET positive results and have been disclosed results, were contacted within 72 hours following Visit 3, to evaluate their overall well-being.

## Cognitive Cohort Classification

The 930 participants were classified by the Biohermes study team into three cohorts: cognitively unimpaired (CU; n=398), mild cognitive impairment (MCI; n=291), or probable Alzheimer’s disease-related dementia (ADRD; n=241) was based on the following criteria:

1. ***Cohort 1, Cognitively Unimpaired:***
   1. No reported memory loss or concerns (as reported by participant and study partner)
   2. Mini-Mental Status Exam (MMSE) score of 25 to 30 inclusive
   3. Rey Auditory Verbal Learning Test (RAVLT)- Delayed Recall Score within normal range based on age-adjusted mean
   4. In the investigator’s judgment, no evidence of functional decline based on the Functional Activities Questionnaire (FAQ) score/study partner report
2. ***Cohort 2, MCI:***
   1. A diagnosis of Mild Cognitive Impairment (MCI) given within 3 months of Visit 1 and verified through medical records

OR

- 1. All the following criteria:
     1. Memory loss may or may not be reported by participant or study partners
     2. MMSE score of 24-30 inclusive
     3. RAVLT- Delayed Recall Score of 1 standard deviation below the age-adjusted mean
     4. In the investigator’s judgment, no evidence of functional decline based on the FAQ score/study partner report

1. ***Cohort 3, probable ADRD:***
   1. A diagnosis of probable Alzheimer’s disease based on the National Institute of Aging (NIA) criteria given within 3 months of Visit 1 and verified through medical records

OR

- 1. All the following criteria:
     1. Reported memory loss by participant or study partner
     2. MMSE score of 20-26
     3. RAVLT- Delayed Recall Score of 1 standard deviation below the age-adjusted mean
     4. In the investigator’s judgment, evidence of functional decline based on FAQ score/study partner report

It is important to note that both MMSE and RAVLT were used for cohort definition, which also considered FAQ information and clinical impression by the investigator at baseline. All analyses that consider the classification performance of MMSE and RAVLT for cognitive impairment are biased due to this circularity.

##

## Cognitive Assessments

The MMSE is a 30-point test commonly used to screen for cognitive impairment in elderly adults and assesses orientation, memory, attention, naming, comprehension, verbal, and written commands. Reportedly, the MMSE can detect mild to moderate stages of dementia with acceptable sensitivity and specificity, but can take 10–15 minutes to complete, especially in individuals with dementia.

The RAVLT is a verbal list-learning test widely used in clinical and research settings to assess verbal memory including encoding, learning, storage, consolidation, and subsequent free recall or recognition. Delayed recall is completed after approximately 25 minutes.

The Linus Health DCR is an FDA-listed Class II 510k exempt software as a medical device, and a digital and AI-enabled adaptation of the well-known, paper-based Mini-Cog. The DCR detects signs of cognitive impairment by analyzing the individual’s performance using a combination of a digital clock drawing test (DCTclock™) and 3-word immediate and delayed verbal recall tests. This multimodal digital assessment captures drawing and voice process metrics and employs automated scoring and AI algorithms to provide objective insights into various cognitive domains including verbal memory, executive function, visuospatial reasoning, and motor function.

##

## Supplementary Results


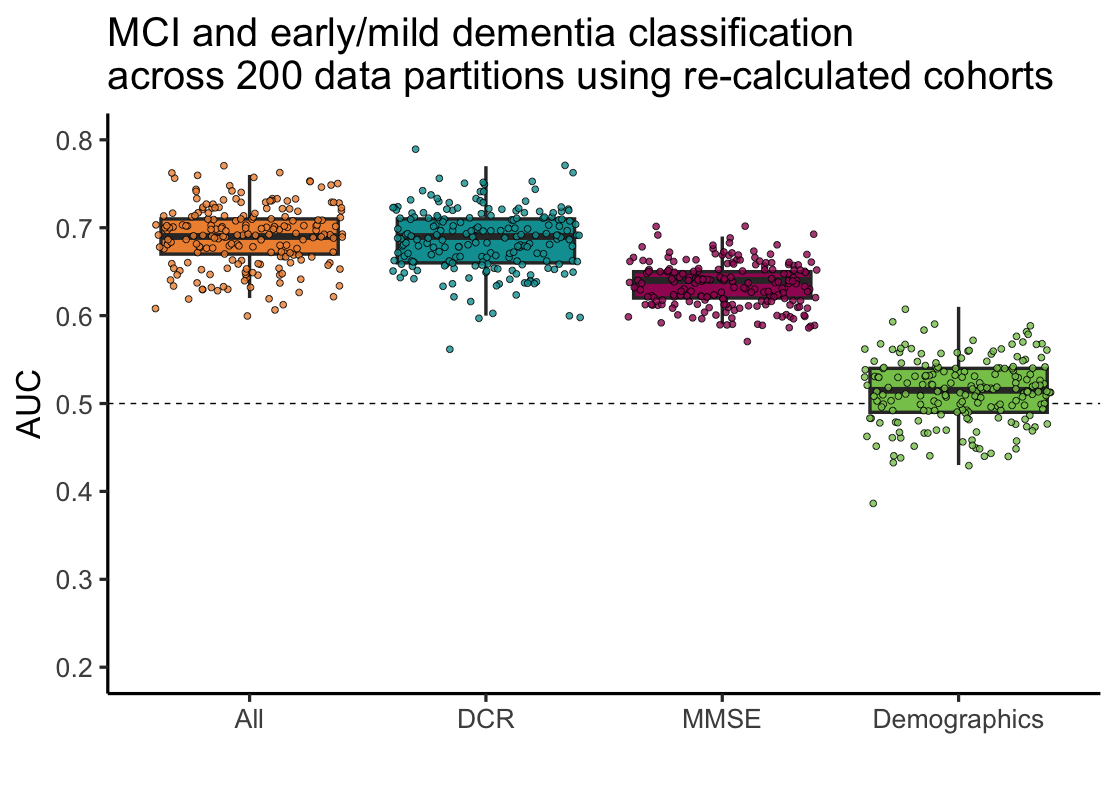


**Supplementary Figure 1:** Distribution of Area Under the receiver operating characteristic Curve (AUC) for each model based on a 200-iteration bootstrapped procedure. This version uses a more stringent RAVLT threshold of -1.5 SD in order to determine amnestic components of MCI. Relative differences are the same as in the results using the original -1 SD RAVLT threshold.

| Model | Sensitivity | Specificity | PPV | NPV | AUC |
| --- | --- | --- | --- | --- | --- |
| DCR | 0.49 (0.05) | 0.75 (0.04) | 0.58 (0.04) | 0.67 (0.02) | 0.69 (0.03) |
| MMSE | 0.57 (0.04) | 0.69 (0.03) | 0.68 (0.02) | 0.59 (0.02) | 0.64 (0.02) |
| Demographics | 0.47 (0.06) | 0.54 (0.05) | 0.43 (0.03) | 0.59 (0.02) | 0.51 (0.03) |

**Supplementary Table 1:** Classification performance per test using cohorts with a RAVLT threshold of -1 SD. PPV = positive predictive value; NPV = negative predictive value; AUC = area under the receiver operating characteristic curve.
